# Knowledge and attitudes toward anaphylaxis to local anesthetics in dental practice

**DOI:** 10.1038/s41405-024-00210-x

**Published:** 2024-04-04

**Authors:** Ivan Cherrez-Ojeda, Juan C. Gallardo-Bastidas, Gabriela Rouillon Borrero, Hans Mautong, Paola Andrea Mena Silva, Zouina Sarfraz, Azza Sarfraz, Leonardo Cano, Karla Robles-Velasco

**Affiliations:** 1 Universidad Espíritu Santo, Samborondón, 0901952 Ecuador; 2 Respiralab, Respiralab Research Group, Guayaquil, Ecuador; 3 Universidad Católica de Santiago de Guayaquil Carrera de Odontología, Guayaquil, 09-01-4671 Ecuador; 4 Universidad Regional Autónoma de Los Andes UNIANDES, Ambato, 180215 Ecuador; 5 Department of Research and Publications, Fatima Jinnah Medical University, Lahore, PB Pakistan; 6 Department of Pediatrics and Child Health, Aga Khan University, Stadium Road, P.O Box 3500, Karachi, 74800 Pakistan

**Keywords:** Dental education, Dental public health

## Abstract

**Objective/aim:**

The absence of a comprehensive understanding of potential anaphylactic reactions to local anesthetics (LAs) and management can result in grave consequences. For this reason we aim to assess Latin American dentists’ knowledge, preparedness, and competency in managing anaphylactic reactions to LAs.

**Materials and Methods:**

**Design:** A cross-sectional study was conducted from November 2021 to February 2022. Board-certified dentists answered a survey comprising 26 structured questions. Chi-square tests and logistic regression models were performed in Stata 17.0. **Setting:** Argentina, Brazil, Colombia, Costa Rica, Ecuador, Honduras, Mexico, Peru, Venezuela, and other Latin American countries.

**Results:**

Of 507 respondents, lidocaine was the most frequently used LA (88.1%). While 85.2% could identify dyspnea as a symptom of anaphylaxis, only 50.1% knew the correct route for epinephrine administration, and just 43.5% had epinephrine in their emergency kits. Confidence in managing anaphylactic reactions was low (9.6%). Older age was inversely related to both knowledge of anaphylaxis management and the possession of epinephrine (*P* = 0.003 and *P* = 0.0001, respectively).

**Discussion:**

Our study highlights a concerning discrepancy between the practical readiness of Latin American dentists in handling anaphylaxis.

**Conclusion:**

The study’s findings underscore the need for educational interventions to improve the readiness to identify and handle anaphylactic emergencies in dental practice.

## Introduction

In routine clinical settings, dental practitioners administer a variety of pharmacological substances, including but not limited to analgesics, antibiotics, antifungals, and local anesthetics (LAs) [1]. Notably, LAs play a pivotal role in mitigating pain and discomfort, thus enabling the proficient completion of diverse dental procedures such as restorations, scaling and root planning, endodontics, and minor surgeries [2–4].

LAs function by blocking sodium ion channels in neuronal membranes, which disrupts neural signal transmission and results in localized anesthesia [2, 5]. These agents are generally categorized into esters and amides based on their chemical structures [6]. Within the dental domain, amide-based anesthetics—namely, lidocaine, mepivacaine, bupivacaine, articaine, and prilocaine—are predominantly utilized [5, 6]. Lidocaine is particularly noteworthy for its safety profile and high tolerability, solidifying its status as the preferred LA in contemporary dental practice [7]. Despite its prevalent use, attention must be paid to its pharmacokinetics, contraindications, and potential adverse effects [7–9].

Allergic reactions to LAs, though rare, can be mediated by immune responses to specific chemical components or additives such as metabisulfite and preservatives like methylparaben [8, 9]. Such reactions can manifest as either type IV hypersensitivity (delayed reactions) characterized by allergic dermatitis and swelling, or type I hypersensitivity (immediate reactions) which could be life-threatening, featuring symptoms like bronchospasm, angioedema, and anaphylaxis [7, 10]. These immediate hypersensitive reactions are exceedingly rare, occurring in less than 1% of cases [9].

Management strategies for allergic reactions differ based on their severity. Mild symptoms may be controlled with antihistamines, while severe, multisystemic symptoms necessitate immediate intervention with epinephrine as the first-line treatment [11–13]. Additionally, a spectrum of non-allergic adverse reactions exist, including psychological apprehensions and physiological responses like vasovagal syncope or drug toxicity [7–9].

Remarkably, some dental professionals tend to hastily diagnose anesthetic allergies without thorough clinical evaluation or specialist consultation [14, 15]. Despite the extremely low incidence of true anaphylactic reactions, dental practitioners, particularly those specializing in fields such as oral and maxillofacial surgery, endodontics, and periodontics, should be adequately trained to diagnose and manage such events [2, 3, 7, 9, 16]. The absence of comprehensive understanding in allergy testing, diagnosis, and management can result in grave consequences.

Therefore, the objective of this cross-sectional study is to evaluate the awareness, preparedness, and competency of Latin American dentists concerning anaphylactic reactions to LAs. Specifically, this research aims to assess dental professionals’ knowledge of guidelines and protocols preceding anesthetic administration, their LA preferences, experiences with adverse events, and their competence in managing anaphylactic episodes [8, 9, 13, 16].

## Material and methods

### Study design and data collection

This study employed a cross-sectional design, utilizing a web-based survey platform, QuestionPro, to collect responses. The survey contained 26 structured, multiple-choice questions and targeted board-certified dentists practicing in various Latin American countries (Supplementary File). The data collection period spanned from November 8, 2021, to February 28, 2022.

### Sampling method

A snowball sampling strategy was implemented to disseminate the survey, primarily through social media channels like WhatsApp and via email. An introductory message along with the survey’s URL was sent to prospective participants, who were then encouraged to circulate the survey among their professional contacts.

### Survey structure

After accessing the survey link and providing informed consent, participants proceeded to answer the questions. The survey was designed to take ~8 min to complete and comprised three main sections:

- *Demographic Information:* This section contained 6 questions focused on gathering background information, such as Gender, Sector of Practice, Area of Practice, Type of Dentist, Specialty, and Country.
- *Anaphylaxis Knowledge:* This portion of the survey consists of 13 questions, which were adapted from a prior study carried out in Chennai [3]. The questions delve into various aspects, including the preference for specific LAs, protocols for administration, recognizing signs and symptoms of an anaphylaxis reaction, knowledge about medical management, as well as attitudes towards both anaphylaxis and its treatment options.
- *Attitude Towards Anaphylaxis:* This portion of the survey consists of 5 questions with an aim to discern practitioners’ attitudes towards the significance and management of anaphylaxis. It covers the perceived importance of recognizing anaphylaxis, confidence in identifying at-risk patients, and proficiency in managing and treating these cases.

### Inclusion and exclusion criteria

The only requirement for participation was being a board-certified dentist practicing in Latin America. Dental students, professionals working outside of Latin America, and incomplete responses were excluded from the study.

### Statistical analysis

Data were analyzed using Stata statistical software (version 17.0; Stata Corporation, College Station, Texas, USA). Categorical variables were summarized as frequencies and percentages, while continuous variables were expressed as means ± standard deviations, and appropriate statistical tests were used to compare these. The chi-square test was utilized for comparisons of categorical variables. Both univariate and multivariate logistic regression analyses were conducted to identify potential associations between dependent and independent variables. Variables that achieved statistical significance in the univariate analyses, and showed no signs of collinearity, were incorporated into the multivariate model. A 2-tailed *P* value of less than 0.05 was considered to indicate statistical significance.

### Ethics

All participants were informed of the study’s objectives. An informed consent was completed prior to their voluntary participation in the survey which also declared that their identity would not be revealed. This study was approved by the local ethics committee “Comité de ética e Investigación en Seres Humanos” (CEISH) according to the principles established by the Declaration of Helsinki.

## Results

### Participant demographics

The survey reached a total of 704 Latin American dentists, with 507 respondents completing it, yielding a response rate of 72.0%. Of these, 27 respondents were excluded based on the study criteria: 15 were dental students, and 12 were practicing outside Latin America. Ultimately, data from 480 participants were included in the final analysis.

The mean age of the surveyed population was 35 years, with a standard deviation of 10 years. Females comprised 59.3% of the respondents. Among the participants, general dentists were the most prevalent, making up 49.2% of the sample. The average professional experience among the dentists was ~10 years, with a standard deviation of 9 years.

### Geographic and professional distribution

A large majority of the respondents hailed from Ecuador (81.9%), followed by Colombians (5.4%), Hondurans (4.2%), and Mexicans (2.1%). A smaller proportion of the sample consisted of dentists from Peru, Brazil, Argentina, Venezuela, Costa Rica, and other Latin American countries. Most of the dentists were employed in private practices (66.3%) and located in urban settings (78.2%).

In terms of specialization, 45.0% of the participants were specialists, primarily in prosthodontics and endodontics, each making up 19.2% of this subgroup (Table 1).

**Table 1:** Demographics and professional background of surveyed dental professionals.

Characteristic	Category	Frequency	Percentage, %
Gender	Male	194	40.7%
	Female	283	59.3%
Age, mean (SD)			35 (10)
Years of dental practice, mean (SD)			10 (9)
In which sector do you practice?	Private Practice	318	66.3%
	Public sector	85	17.7%
	Both	77	16%
In what area do you practice?	Urban area	366	78.2%
	Rural area	49	10.5%
	Both	42	9%
	Other	11	2.4%
Type of dentist	General dentist	236	49.2%
	Specialist dentist	216	45%
	Specialty student	28	5.8%
Specialty	Endodontics	41	19.2%
	Orthodontics and orthopedics	33	15.4%
	Periodontics	25	11.7%
	Oral Rehabilitation/Prosthodontics	41	19.2%
	Pediatric dentistry	19	8.9%
	Oral and/or Maxillofacial Surgery	28	13.1%
	Oral pathology	2	0.9%
	Oral implantology	17	7.9%
	Oral radiology	3	1.4%
	Other	5	2.3%
Country	Ecuador	393	81.9%
	Peru	6	1.3%
	Colombia	26	5.4%
	Mexico	10	2.1%
	Costa Rica	1	0.2%
	Honduras	20	4.2%
	Venezuela	4	0.8%
	Bolivia	1	0.2%
	Argentina	2	0.4%
	Brazil	3	0.6%
	Other^a^	14	2.9%

^a^Country Other: Chile, Guatemala, Dominican Republic, Panama.

### Practices in local anesthesia administration

The majority of professionals predominantly used Lidocaine (88.1%) as their first choice for LAs, followed by Mepivacaine (40.8%), and Articaine (29.0%) (Table S1). In terms of adjuvants, a majority (60.2%) preferred LAs combined with epinephrine (Table S1). When it comes to pre-operative preparation, 96.7% of surveyed professionals took a detailed clinical history of their patients before initiating any procedure (Table S1). However, a substantial number of them (79.0%) did not administer a test dose of the local anesthetic prior to the treatment (Table S1).

### Knowledge and awareness about anaphylaxis

Only 21.3% of dentists reported having encountered a systemic adverse reaction triggered by local anesthesia. As for recognizing symptoms of anaphylaxis, 85.2% identified dyspnea as a major clinical manifestation. Although 56.7% knew that epinephrine was the drug of choice for treating anaphylaxis, only 50.1% were aware that the correct route of administration is intramuscular. Further, just 43.5% had epinephrine as part of their emergency medical kit, while antihistamines were more commonly available (53.1%). Interestingly, 22.1% had none of the listed emergency drugs in their offices (Table S1).

### Attitudes toward anaphylaxis

A significant percentage of professionals (63.3%) considered anaphylaxis as extremely important, with 65.4% acknowledging the crucial role of correctly identifying the condition. However, only 28.7% expressed strong confidence in identifying anaphylaxis. Even fewer felt confident managing an anaphylactic reaction (9.6%) or administering epinephrine in such cases (9.4%) (Table 2).

**Table 2:** Attitudes toward anaphylaxis.

Category	Frequency	Percentage, %
1. As a clinical disorder, anaphylaxis is:		
Not important	0	0%
Somewhat important	31	6.5%
Important	46	9.6%
Very important	99	20.6%
Extremely important	304	63.3%
2. Identifying patients with possible anaphylaxis is:		
Not important	0	0%
Somewhat important	16	3.3%
Important	48	10%
Very important	102	21.3%
Extremely important	314	65.4%
3. I feel confident identifying patients at risk of anaphylaxis:		
Strongly disagree	16	3.3%
In disagreement	35	7.3%
Neither agree or disagree	109	22.7%
Agree	182	37.9%
Strongly agree	138	28.7%
4. I am confident in my ability to manage patients with anaphylaxis		
Strongly disagree	25	5.2%
In disagreement	71	14.8%
Neither agree or disagree	164	34.2%
Agree	175	36.5%
Strongly agree	45	9.4%
5. I am confident in my ability to use epinephrine in patients with anaphylaxis.		
Strongly disagree	29	6%
In disagreement	94	19.6%
Neither agree or disagree	153	31.9%
Agree	158	32.9%

### Regional comparison: Ecuador vs. rest of Latin America

Although the majority of respondents were from Ecuador, there were no statistically significant differences in terms of knowledge and attitudes between Ecuador and other Latin American countries (*P* > 0.05). Nevertheless, professionals from Ecuador displayed more confidence in managing patients with anaphylaxis (49.1% vs. 31.1%, *P* = 0.002) and in using epinephrine (45.8% vs. 27.6%, *P* = 0.002).

### Knowledge of drug of choice in anaphylaxis (univariate and multivariate regression analyses)

Our univariate analysis revealed several important findings (Table S2). Notably, respondents who reported having seen a patient with a systemic adverse reaction caused by local anesthesia were 1.48 times more likely to possess knowledge of the drug of choice for anaphylaxis, although this finding was not statistically significant (OR = 1.48, 95% CI = 0.95–2.30, *P* = 0.08).

Being confident in identifying patients at risk of anaphylaxis (OR = 1.11, 95% CI = 0.76–1.62, *P* = 0.602), in managing anaphylaxis patients (OR = 1.05, 95% CI = 0.73–1.50, *P* = 0.805), and in using epinephrine (OR = 1.44, 95% CI = 0.99–2.08, *P* = 0.053) were not strongly associated with better knowledge of the drug of choice in anaphylaxis.

However, recognizing dyspnea as a symptom of anaphylaxis was associated with significantly better knowledge (OR = 1.73, 95% CI = 1.04–2.87, *P* = 0.034).

Other variables, including sector of practice, type of dentist, and preference for local anesthesia with or without epinephrine, did not significantly influence the respondents’ knowledge. Age (OR = 0.97, 95% CI = 0.96–0.99, *P* = 0.003) and years of professional experience (OR = 0.96, 95% CI = 0.94–0.98, *P* = 0.0001) were found to be statistically significant but suggest an inverse relationship with the knowledge level.

In the multivariate regression analysis (Table 3), recognizing dyspnea as a symptom of anaphylaxis was almost significant (OR = 1.62, 95% CI = 0.97–2.72, *P* = 0.068). Age remained a significant predictor of knowledge, with a similar odds ratio to that in the univariate analysis (OR = 0.97, 95% CI = 0.96–0.99, *P* = 0.003).

**Table 3:** Multivariate regression analysis predicting knowledge of drug of choice in anaphylaxis.

Drug of choice/Variable	OR (Odds Ratio)	95% Confidence Interval	*P* value
Symptoms of anaphylaxis: Dyspnea	1.62	0.97–2.72	0.068
Age	0.97	0.96–0.99	0.003

### Possession of epinephrine in office (univariate and multivariate regression analyses)

In the univariate logistic regression analyses (Table S3), several variables were found to be statistically significant predictors for the possession of epinephrine in the office. Practitioners who felt confident in identifying patients at risk of anaphylaxis were more likely to have epinephrine available (OR = 1.57, 95% CI = 1.06–2.32, *P* = 0.023). Similarly, confidence in the ability to manage anaphylaxis patients (OR = 1.87, 95% CI = 1.30–2.69, *P* = 0.001) and use epinephrine in such patients (OR = 2.5, 95% CI = 1.72–3.63, *P* = 0.0001) were strongly associated with the possession of epinephrine.

Understanding cutaneous eruption as a symptom of anaphylaxis was also a significant factor (OR = 1.45, 95% CI = 1.01–2.09, *P* = 0.043). Age (OR = 0.96, 95% CI = 0.94–0.98, *P* = 0.0001) and years of professional experience (OR = 0.96, 95% CI = 0.94–0.98, *P* = 0.0001) were found to be statistically significant but suggest an inverse relationship with the possession of epinephrine.

On the other hand, having witnessed a patient with a systemic adverse reaction caused by local anesthesia (OR = 0.83, 95% CI = 0.54–1.29, *P* = 0.42), recognizing dyspnea (OR = 1.14, 95% CI = 0.68–1.90, *P* = 0.62) or hypotension (OR = 1.15, 95% CI = 0.79–1.68, *P* = 0.472) as symptoms of anaphylaxis, and routinely administering a test dose (OR = 0.74, 95% CI = 0.47–1.14, *P* = 0.175) were not statistically significant predictors.

In the multivariate logistic regression analyses (Table 4), confidence in the ability to use epinephrine in patients with anaphylaxis remained a highly significant predictor (OR = 2.84, 95% CI = 1.68–4.80, *P* = 0.0001). Age continued to be a significant but inverse predictor (OR = 0.96, 95% CI = 0.94–0.98, *P* = 0.0001). However, confidence in identifying patients at risk of anaphylaxis (OR = 1.23, 95% CI = 0.77–1.94, *P* = 0.386) and in managing anaphylaxis patients (OR = 0.99, 95% CI = 0.58–1.69, *P* = 0.964) were not significant in the multivariate model.

**Table 4:** Multivariate logistic regression analyses predicting the possession of epinephrine in office.

Drug of choice/Variable	OR (Odds Ratio)	95% Confidence Interval	*P* value
I feel confident identifying patients at risk of anaphylaxis	1.23	0.77–1.94	0.386
I am confident in my ability to manage anaphylaxis patients	0.99	0.58–1.69	0.964
I am confident in my ability to use epinephrine in patients with anaphylaxis	2.84	1.68–4.80	0.0001
Symptoms of anaphylaxis: Cutaneous eruption	1.43	0.96–2.11	0.075
Age	0.96	0.94–0.98	0.0001

### Confident attitude in identifying patients at risk of anaphylaxis (univariate and multivariate regression analyses)

In the univariate logistic regression analysis (Table S4), practitioners who have seen a patient with a systemic adverse reaction due to local anesthesia had lower odds of being confident in identifying patients at risk of anaphylaxis (OR = 0.51, 95% CI = 0.31–0.85, *P* = 0.01). On the other hand, higher odds of confidence were observed among practitioners who were older (OR = 1.03, 95% CI = 1.01–1.05, *P* = 0.003), male (OR = 1.87, 95% CI = 1.26–2.81, *P* = 0.002), or had more years of professional experience (OR = 1.03, 95% CI = 1.01–1.06, *P* = 0.003).

Other variables such as recognizing symptoms of anaphylaxis like cutaneous eruption (OR = 1.42, 95% CI = 0.97–2.09, *P* = 0.071), dyspnea (OR = 1.37, 95% CI = 0.81–2.29, *P* = 0.238), or hypotension (OR = 1.01, 95% CI = 0.68–1.51, *P* = 0.946) were not statistically significant predictors. Similarly, other aspects such as the sector or area of practice, type of dentist, and preferences for local anesthesia did not significantly predict the confidence in identifying patients at risk of anaphylaxis.

In the multivariate logistic regression analysis (Table 5), the odds of being confident in identifying patients at risk of anaphylaxis were significantly associated with age (OR = 1.02, 95% CI = 1.01–1.05, *P* = 0.029) and sex (OR = 1.75, 95% CI = 1.15–2.67, *P* = 0.009). Interestingly, having seen a patient with a systemic adverse reaction due to local anesthesia was not a significant predictor in the multivariate model (OR = 0.69, 95% CI = 0.40–1.18, *P* = 0.178).

**Table 5:** Multivariate logistic regression analyses predicting a confident attitude in identifying patients at risk of anaphylaxis.

Drug of choice/Variable	OR (Odds Ratio)	95% Confidence Interval	*P* value
Have you ever seen a patient with a systemic adverse reaction caused by local anesthesia?	0.69	0.40–1.18	0.178
Age	1.02	1.01–1.05	0.029
Sex	1.75	1.15–2.67	0.009

## Discussion

Our survey received a robust 72% response rate, predominantly from Ecuadorian dentists, resulting in a final participant count of 480. The surveyed population had a mean age of 35 years, and 59.3% were females. Despite 63.3% considering anaphylaxis extremely important, only 28.7% expressed strong confidence in identifying it. Moreover, 88.1% preferred Lidocaine as their local anesthetic, but a striking 79% did not administer a test dose prior to treatment. Although 85.2% could identify dyspnea as a symptom of anaphylaxis, just 50.1% were aware that intramuscular epinephrine is the correct route for anaphylaxis management. Age and gender emerged as significant predictors of confidence, while prior experience with adverse reactions was not a strong determinant. These findings point to a significant disconnect between the importance assigned to anaphylaxis management and actual preparedness among Latin American dentists.

Our study highlights a concerning discrepancy between the perceived importance and practical readiness of Latin American dentists in handling anaphylaxis, a life-threatening hypersensitivity reaction as delineated by the WAO Anaphylaxis Committee [17]. Although LAs are routinely used in dental procedures and are generally deemed safe, anaphylaxis can still occur and present as a critical clinical hazard.

When considering patient history and allergies to anesthetics, our data reveal that 35.2% of surveyed dentists would prefer to refer patients with a history of allergies to an allergy specialist, while 25% would choose to discontinue the procedure altogether. These findings are in stark contrast to previous studies where ~70% of dentists would either halt the procedure or refer to a specialist [3, 16, 18]. Moreover, unlike studies from Chennai and Iran, where witnessing anaphylaxis was more common, most respondents in our survey had never encountered an adverse reaction associated with LAs. These observations echo only a study from Turkey [3, 18, 19].

Interestingly, prior exposure to adverse reactions did not confer increased confidence in managing future episodes of anaphylaxis. This suggests that dentists who have encountered anaphylaxis before may become acutely aware of their lack of adequate training to deal with such events effectively, thereby highlighting the need for enhanced educational measures.

In terms of diagnosis, dyspnea was most frequently identified as an indicative symptom of anaphylaxis, which aligns well with existing literature [3, 18, 20]. Furthermore, those who correctly identified dyspnea was also more likely to be aware that epinephrine is the drug of choice for treating anaphylaxis. However, a mere 56.7% of respondents correctly identified epinephrine as the preferred treatment, a statistic that lags behind several other studies [3, 18, 19].

Within the subgroup of oral and maxillofacial surgeons, 28% felt confident in their ability to manage anaphylaxis and administer epinephrine, corroborating findings from both India and Brazil [20, 21]. Alarmingly, a notable fraction (30%) would choose intravenous administration of epinephrine, a route associated with a substantially higher risk of overdose and adverse cardiovascular effects than intramuscular administration [22].

Despite the majority (63.3%) acknowledging the clinical severity of anaphylaxis, only 9.4% felt fully equipped to manage it. Many opted for antihistamines and glucocorticoids in their emergency kits, which while useful for other medical conditions, are not sufficient to counteract anaphylactic shock [23]. This reveals a critical gap in emergency preparedness and indicates a deficiency in the understanding of pharmacology among dentists.

Ecuadorian dentists reported greater confidence compared to their counterparts in the rest of Latin America, both in managing anaphylaxis (49.1% vs. 31.1%) and using epinephrine (45.8% vs. 27.6%). These regional differences warrant further investigation and may offer insights for targeted educational interventions. Finally, while considerable literature exists on emergency management guidelines in the dental care sector, the countries examined do not have any federal rules that provide a mandatory minimum standard for the operation of dental offices. However, in this particular situation, it is crucial to emphasize the necessity of laws implemented by state organizations to prevent unforeseen incidents that may arise due to insufficient equipment or training.

### Limitations

Our study is subject to several limitations that merit consideration. Primarily, the data predominantly originate from Ecuadorian dentists, which raises questions about the generalizability of our findings across the broader Latin American region. Secondly, the survey respondents were informed about the study’s aim, a factor that could introduce response bias, thereby potentially influencing the veracity of the reported results. Another critical limitation is that the survey instrument utilized for data collection has not been formally validated. This lack of validation poses a challenge for the future replicability of our findings. Despite these limitations, the strengths of our study include a substantial sample size and a participant pool that reflects diverse demographic characteristics, enhancing the robustness and potential applicability of our data.

## Conclusion

While adverse reactions during the administration of anesthesia during dental procedures are relatively rare, they possess the potential to be life-threatening, specifically in the case of anaphylaxis. Given the critical nature of such events, our study highlights the imperative need for ongoing educational initiatives aimed at equipping dentists with the requisite knowledge and skills for diagnosing and managing anaphylactic reactions effectively. This enhanced training would not only elevate the standard of patient care but also contribute to better preparedness among dental professionals for handling medical emergencies.

### Supplementary information

Supplementary Information

## Data Availability

The datasets used and/or analyzed during the current study available from the corresponding author on reasonable request.

## References

[1] Ganda K. Dentist’s guide to medical conditions, medications and complications. Wiley; 2013. https://books.google.com.pa/books?id=YGdlUNRLJJUC.

[2] Janas-Naze A, Osica P (2019). The incidence of lidocaine allergy in dentists: an evaluation of 100 general dental practitioners. Int J Occup Med Environ Health.

[3] Krishnamurthy M, Venugopal NK, Leburu A, Kasiswamy Elangovan S, Nehrudhas P (2018). Knowledge and attitude toward anaphylaxis during local anesthesia among dental practitioners in Chennai–a cross-sectional study. Clin Cosmet Investig Dent.

[4] Tomoyasu Y, Mukae K, Suda M, Hayashi T, Ishii M, Sakaguchi M (2011). Allergic reactions to local anesthetics in dental patients: analysis of intracutaneous and challenge tests. Open Dent J.

[5] Becker DE, Reed KL (2012). Local anesthetics: review of pharmacological considerations. Anesth Prog.

[6] Decloux D, Ouanounou A (2021). Local anaesthesia in dentistry: a review. Int Dent J.

[7] Bahar E, Yoon H (2021). Lidocaine: a local anesthetic, its adverse effects and management. Medicina.

[8] Bina B, Hersh EV, Hilario M, Alvarez K, McLaughlin B (2018). True allergy to amide local anesthetics: a review and case presentation. Anesth Prog.

[9] Cherobin ACFP, Tavares GT (2020). Safety of local anesthetics. An Bras Dermatol.

[10] Speca SJ, Boynes SG, Cuddy MA (2010). Allergic reactions to local anesthetic formulations. Dent Clin.

[11] Takazawa T, Yamaura K, Hara T, Yorozu T, Mitsuhata H, Morimatsu H (2021). Practical guidelines for the response to perioperative anaphylaxis. J Anesth.

[12] Kroigaard M, Garvey LH, Gillberg L, Johansson SGO, Mosbech H, Florvaag E (2007). Scandinavian Clinical Practice Guidelines on the diagnosis, management and follow‐up of anaphylaxis during anaesthesia. Acta Anaesthesiol Scand.

[13] Frew AJ (2011). What are the ‘ideal’features of an adrenaline (epinephrine) auto‐injector in the treatment of anaphylaxis?. Allergy.

[14] Robertson DP, Keys W, Rautemaa-Richardson R, Burns R, Smith AJ (2015). Management of severe acute dental infections. BMJ.

[15] Needleman I (2016). The good practitioner’s guide to periodontology. Br Soc Periodontol.

[16] Malinovsky JM, Chiriac AM, Tacquard C, Mertes PM, Demoly P (2016). Allergy to local anesthetics: reality or myth?. Presse Méd.

[17] Turner PJ, Worm M, Ansotegui IJ, El-Gamal Y, Rivas MF, Fineman S (2019). Time to revisit the definition and clinical criteria for anaphylaxis?. World Allergy Organ J.

[18] Cetinkaya F, Sezgin G, Aslan OM (2011). Dentists’ knowledge about anaphylaxis caused by local anaesthetics. Allergol Immunopathol.

[19] Eskandari N, Nekourad M, Bastan R (2014). The awareness of anaphylaxis reaction to local anesthesia in Dentistry. J Allergy Asthma.

[20] Soysa NS, Soysa IB, Alles N (2019). Efficacy of articaine vs lignocaine in maxillary and mandibular infiltration and block anesthesia in the dental treatments of adults: A systematic review and meta‐analysis. J Investig Clin Dent.

[21] PINHEIRO AC, MARQUES JF, VIEIRA MS, BRANCO-DE-ALMEIDA LS (2015). Dentists’ knowledge regarding signs and symptoms of the systemic toxicity of local anesthetic solutions. RGO-Rev Gaúcha de Odontol.

[22] Campbell RL, Bellolio MF, Knutson BD, Bellamkonda VR, Fedko MG, Nestler DM (2015). Epinephrine in anaphylaxis: higher risk of cardiovascular complications and overdose after administration of intravenous bolus epinephrine compared with intramuscular epinephrine. J Allergy Clin Immunol Pract.

[23] Baluga JC, Casamayou R, Carozzi E, Lopez N, Anale R, Borges R (2002). Allergy to local anaesthetics in dentistry. Myth or reality?. Allergol Immunopathol.

