# Supplementary material for: Knowledge and attitudes toward anaphylaxis to local anesthetics in dental practice
Source: BDJ Open. 2024 Apr 4;10:28. doi: 10.1038/s41405-024-00210-x (PMC10994913; doi:10.1038/s41405-024-00210-x)
Supplement: Supplementary file 1 — Supplementary Information [file 41405_2024_210_MOESM1_ESM.pdf]

Supplementary Materials

Questionnaire

a) Age of the dentist (years):
   a. 25–30
   b. 31–45
   c. >46

b) Duration of dental practice (years):
   a. Less than 5
   b. 6–15
   c. >16

c) You are qualified as a:
   a. General dentist
   b. Specialist dentist

d) Please write down the names of the local anesthetics that you use in your daily practice:
   a. Lidocaine
   b. Articaine
   c. Prilocaine
   d. Others

e) Do you prefer local anesthesia with adrenaline or without adrenaline?
   a. With adrenaline
   b. Without adrenaline

f) Do you ask your patients if they have any drug allergies before the treatment?
   a. Yes
   b. No

g) Do you ask your patients whether they got local anesthetic administration done prior for any dental procedures?
   a. Yes
   b. No

h) Do you give a test dose on a routine basis?
   a. Yes
   b. No

i) How do you behave when you see a patient with a suspected local anesthesia allergy? a) I do not begin the treatment
   a. I make a skin prick test with the suspected drug by myself
   b. I refer the patient to an allergy specialist with the suspected drug for testing
   c. I treat the patient without local anesthesia
   d. Others

j) How do you behave when you see a patient without any drug allergy, but with any other allergic diseases, such as asthma or hay fever?
   a. I make the treatment without considering the other allergic problems
   b. I accept these problems as a risk factor and refer the patient to the specialist

k) Have you ever seen a patient with systemic adverse reaction due to local anesthesia?
   a. Yes
   b. No

l) If you have seen a case with systemic adverse reaction due to local anesthesia, has this occurred during your treatment?
   a. Yes
   b. No

m) Which symptoms below remind you of anaphylaxis during your treatment?

a. Nausea and vomiting
b. Shortness of breath
c. Skin rash
d. Skin swelling
e. Hypotension

n) What reaction do you suspect suggestive of anaphylaxis after test dose?
a. Skin rashes
b. Itching
c. Dyspnea
d. Sudden fainting

o) Which one of the drugs below do you keep in your office?
a. Epinephrine
b. Antihistamine
c. Corticosteroids
d. Glucagon
e. Salbutamol
f. None of the above

p) Which drug should be used as the first choice in management of anaphylaxis?
a. Epinephrine
b. Antihistamine
c. Corticosteroids
d. Glucagon
e. Salbutamol

q) Which route do you prefer as initial route for epinephrine injection?
a. Intramuscular
b. Subcutaneous
c. Intravenous
d. I do not know

These are not "correct or incorrect" answers; they reflect the respondents' views or practices. However, there are some answers that align more closely with current medical guidelines. Below are those that generally align with these guidelines for the knowledge and attitude component of our survey:

1. **What local anesthetic do you use?**
    - Lidocaine is commonly used, but all listed options are legitimate depending on the situation.
2. **Do you prefer local anesthetics with epinephrine or without epinephrine?**
    - "With Epinephrine" is commonly used to prolong the duration of anesthesia and reduce bleeding, but both have their places.
3. **Do you complete a clinical history of your patients before a procedure or treatment?**
    - "Yes" aligns with best practices for patient safety.
4. **Do you routinely administer a test dose of local anesthetic?**
    - Practices may vary, but administering a test dose is generally not a standard practice for local anesthetics.
5. **How do you behave when you care for a patient with a suspected allergy to local anesthesia?**
    - Referring the patient to an allergist is often considered the best practice.
6. **How do you behave when caring for a patient who does not have drug allergies, but has other allergic conditions, such as asthma or allergic rhinitis?**
    - Considering these problems as a risk factor and possibly referring the patient to a specialist aligns with a cautious approach.
7. **Have you ever seen a patient with a systemic adverse reaction caused by local anesthesia?**
    - This is a yes/no question, so there is no "correct" answer.
8. **If you have seen a case of systemic adverse reaction caused by local anesthesia, did this occur during your treatment?**
    - This is also a yes/no question, so there is no "correct" answer.
9. **What are the symptoms of an anaphylactic reaction during the application of local anesthetics?**
    - Dyspnea, Cutaneous Eruption, Edema, and Hypotension are generally associated with anaphylaxis.
10. **After administering the test dose, what reaction is suspected of anaphylaxis?**

- Dyspnea, Cutaneous reaction, and Hypotension are generally more associated with anaphylaxis than Syncope or Pruritus.

11. **Which of the following medications do you have in your office?**
    - Epinephrine, Antihistamines, and Corticosteroids are often recommended for emergency situations.
12. **What drug should be used as the first option to treat anaphylaxis?**
    - Epinephrine is the first-line treatment for anaphylaxis.
13. **Which route do you prefer for the initial administration of epinephrine injection?**
    - Intramuscular is the recommended route for the initial administration of epinephrine for anaphylaxis.

Note that medical guidelines can change over time and may vary by jurisdiction. Always refer to the most current guidelines and consult medical professionals for the most accurate and personalized advice.

Table S1. Knowledge and Awareness Towards Anaphylaxis.

Characteristic	Category	Frequency	Percentage, %
**1. What local anesthetic do you use?**			
	Lidocaine	423	88.1%
	Articaine	139	29%
	Prilocaine	6	1.3%
	Mepivacaine	196	40.8%
	Bupivacaine	9	1.9%
**2. Do you prefer local anesthetics with epinephrine or without epinephrine?**			
	With Epinephrine	289	60.2%
	Without Epinephrine	37	7.7%
	Both	154	32.1%
**3. Do you complete a clinical history of your patients before a procedure or treatment?**			
	Yes	464	96.7%
	No	16	3.3%
**4. Do you routinely administer a test dose of local anesthetic?**			
	Yes	101	21%
	No	379	79%
**5. How do you behave when you care for a patient with a suspected allergy to local anesthesia?**			
	I do not start the treatment	120	25%
	I perform a skin test with the suspected drug	102	21.3%
	I refer the patient to an allergist for a test with the suspected drug	169	35.2%
	Start treatment without local anesthesia	44	9.2%
	Other	45	9.4%
**6. How do you behave when caring for a patient who does not have drug allergies, but has other allergic conditions, such as asthma or allergic rhinitis?**			
	I perform the treatment without considering the other allergic problems	250	52.1%
	I consider these problems as a risk factor and refer the patient to a specialist.	230	47.9%
**7. Have you ever seen a patient with a systemic adverse reaction caused by local anesthesia?**			
	Yes	102	21.3%
	No	378	78.8%
**8. If you have seen a case of systemic adverse reaction caused by local anesthesia, did this occur during your treatment?**			
	Yes	54	52.9%
	No	48	47.1%
**9. What are the symptoms of an anaphylactic reaction during the application of local anesthetics?**			
Nausea and vomiting	No	378	78.8%
	Yes	102	21.3%
Dyspnea	No	71	14.8%
	Yes	409	85.2%

Cutaneous Eruption	No	248	51.7%
	Yes	232	48.3%
Edema	No	321	66.9%
	Yes	159	33.1%
Hypotension	No	314	65.4%
	Yes	166	34.6%
**10. After administering the test dose, what reaction is suspected of anaphylaxis?**			
Cutaneous reaction	No	217	45.2%
	Yes	263	54.8%
Pruritus	No	263	54.8%
	Yes	217	45.2%
Dyspnea	No	288	60%
	Yes	192	40%
Syncope	No	407	84.8%
	Yes	73	15.2%
**11. Which of the following medications do you have in your office?**			
Epinephrine	No	271	56.5%
	Yes	209	43.5%
Antihistamines	No	225	46.9%
	Yes	255	53.1%
Corticosteroids	No	256	53.3%
	Yes	224	46.7%
Glucagon	No	456	95%
	Yes	24	5%
Albuterol	No	417	86.9%
	Yes	63	13.1%
None	No	374	77.9%
	Yes	106	22.1%
**12. What drug should be used as the first option to treat anaphylaxis?**			
	Epinephrine	272	56.7%
	Antihistaminic	115	24%
	Corticosteroids	84	17.5%
	Glucagon	1	0.2%
	Albuterol	8	1.7%
**13. Which route do you prefer for the initial administration of epinephrine injection?**			
	Intramuscular	240	50.1%
	Subcutaneous	49	10.2%
	Intravenous	119	24.8%
	Don´t know	71	14.8%

Table S2. Univariate Analysis Predicting Knowledge of Drug of Choice in Anaphylaxis.

**Drug of choice/Variable**	**OR (Odds Ratio)**	**95% Confidence Interval**	**P value**
Have you ever seen a patient with a systemic adverse reaction caused by local anesthesia?	1.48	0.95-2.30	0.08
I feel confident identifying patients at risk of anaphylaxis	1.11	0.76-1.62	0.602
I am confident in my ability to manage anaphylaxis patients	1.05	0.73-1.50	0.805
I am confident in my ability to use epinephrine in patients with anaphylaxis	1.44	0.99-2.08	0.053
Symptoms of anaphylaxis: Cutaneous eruption	1.21	0.84-1.73	0.308
Symptoms of anaphylaxis: Dyspnea	1.73	1.04-2.87	0.034
Symptoms of anaphylaxis: Hypotension	1.2	0.82-1.76	0.34
Reaction suspected after test dose: Syncope	1.04	0.63-1.73	0.871
Sector of practice	0.98	0.77-1.24	0.861
Area of practice	1.05	0.82-1.34	0.711
Type of dentist	0.91	0.67-1.23	0.527
Preference for LA with/without epinephrine	0.86	0.70-1.04	0.121
Routinely administer a test dose	1.12	0.72-1.74	0.614
Age	0.97	0.96-0.99	0.003
Sex	0.87	0.60-1.26	0.46
Years of professional experience	0.96	0.94-0.98	0.0001

Table S3. Univariate Logistic Regression Analyses Predicting the Possession of Epinephrine in Office

**Drug of choice/Variable**	**OR (Odds Ratio)**	**95% Confidence Interval**	**P value**
Have you ever seen a patient with a systemic adverse reaction caused by local anesthesia?	0.83	0.54-1.29	0.42
I feel confident identifying patients at risk of anaphylaxis	1.57	1.06-2.32	0.023
I am confident in my ability to manage anaphylaxis patients	1.87	1.30-2.69	0.001
I am confident in my ability to use epinephrine in patients with anaphylaxis	2.5	1.72-3.63	0.0001
Symptoms of anaphylaxis: Cutaneous eruption	1.45	1.01-2.09	0.043
Symptoms of anaphylaxis: Dyspnea	1.14	0.68-1.90	0.62
Symptoms of anaphylaxis: Hypotension	1.15	0.79-1.68	0.472
Reaction suspected after test dose: Syncope	0.83	0.50-1.38	0.476
Sector of practice	1.11	0.87-1.41	0.398
Area of practice	1.18	0.92-1.51	0.182
Type of dentist	0.84	0.62-1.14	0.256
Preference for LA with/without epinephrine	0.87	0.71-1.06	0.155
Routinely administer a test dose	0.74	0.47-1.14	0.175
Age	0.96	0.94-0.98	0.0001
Sex	1.26	0.87-1.82	0.221
Years of professional experience	0.96	0.94-0.98	0.0001

Table S4. Univariate Logistic Regression Analyses Predicting a Confident Attitude in Identifying Patients at Risk of Anaphylaxis

Drug of choice/Variable	OR (Odds Ratio)	95% Confidence Interval	P value
Have you ever seen a patient with a systemic adverse reaction caused by local anesthesia?	0.51	0.31-0.85	0.01
Symptoms of anaphylaxis: Cutaneous eruption	1.42	0.97-2.09	0.071
Symptoms of anaphylaxis: Dyspnea	1.37	0.81-2.29	0.238
Symptoms of anaphylaxis: Hypotension	1.01	0.68-1.51	0.946
Reaction suspected after test dose: Syncope	0.95	0.56-1.61	0.857
Sector of practice	0.96	0.75-1.24	0.765
Area of practice	1.3	0.98-1.73	0.067
Type of dentist	1.07	0.78-1.47	0.668
Preference for LA with/without epinephrine	0.96	0.78-1.18	0.673
Routinely administer a test dose	0.63	0.38-1.04	0.07
Age	1.03	1.01-1.05	0.003
Sex	1.87	1.26-2.81	0.002
Years of professional experience	1.03	1.01-1.06	0.003
